# Effects of Acidity on Reactive Oxygen Species Formation from Secondary Organic Aerosols

**DOI:** 10.1021/acsenvironau.2c00018

**Published:** 2022-04-29

**Authors:** Jinlai Wei, Ting Fang, Manabu Shiraiwa

**Affiliations:** Department of Chemistry, University of California, Irvine, Irvine, California 92697-2025, United States

**Keywords:** pH, secondary organic aerosols, reactive oxygen species, organic hydroperoxides, quinones, electron paramagnetic resonance

## Abstract

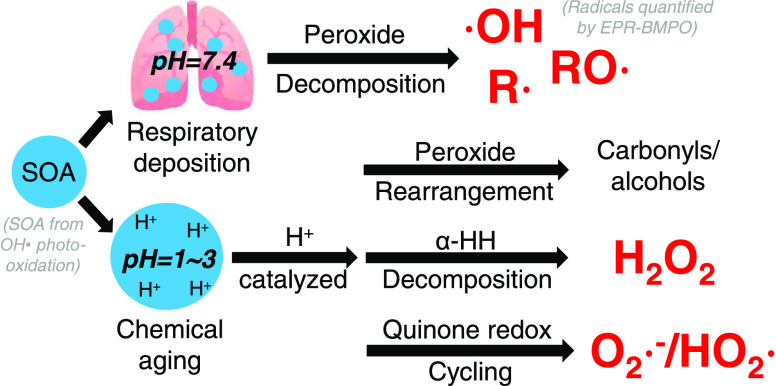

Reactive oxygen species (ROS) play a critical role in the chemical transformation of atmospheric secondary organic aerosols (SOA) and aerosol health effects by causing oxidative stress *in vivo*. Acidity is an important physicochemical property of atmospheric aerosols, but its effects on the ROS formation from SOA have been poorly characterized. By applying the electron paramagnetic resonance spin-trapping technique and the Diogenes chemiluminescence assay, we find highly distinct radical yields and composition at different pH values in the range of 1–7.4 from SOA generated by oxidation of isoprene, α-terpineol, α-pinene, β-pinene, toluene, and naphthalene. We observe that isoprene SOA has substantial hydroxyl radical (^•^OH) and organic radical yields at neutral pH, which are 1.5–2 times higher compared to acidic conditions in total radical yields. Superoxide (O_2_^•–^) is found to be the dominant species generated by all types of SOAs at lower pH. At neutral pH, α-terpineol SOA exhibits a substantial yield of carbon-centered organic radicals, while no radical formation is observed by aromatic SOA. Further experiments with model compounds show that the decomposition of organic peroxide leading to radical formation may be suppressed at lower pH due to acid-catalyzed rearrangement of peroxides. We also observe 1.5–3 times higher molar yields of hydrogen peroxide (H_2_O_2_) in acidic conditions compared to neutral pH by biogenic and aromatic SOA, likely due to enhanced decomposition of α-hydroxyhydroperoxides and quinone redox cycling, respectively. These findings are critical to bridge the gap in understanding ROS formation mechanisms and kinetics in atmospheric and physiological environments.

## Introduction

Secondary organic aerosols (SOA) account for a substantial fraction of atmospheric particulate matter (PM) and play a critical role in climate, air quality, and public health.^1,2^ SOA originate from the multigenerational oxidation of volatile organic compounds (VOCs), followed by nucleation and condensation of the oxidation products.^3^ Acidity is a key physicochemical property of atmospheric PM and droplets, influencing numerous atmospheric and environmental processes, including gas-particle partitioning,^4^ organic aerosol composition and reactivity,^5,6^ cloud processing,^7^ and nutrient availability in terrestrial and marine ecosystems.^8,9^ The atmospheric aerosols and droplets exhibit a wide range of pH, ranging from highly acidic (−1 to 2) in sulfate-rich aerosols^10−12^ to near-neutral (5–7) in sea-salt particles, dust, and cloud droplets.^13−15^ Acidity impacts multiphase chemical processes in atmospheric waters including the uptake of acidic or basic compounds and the phase partitioning and composition of aerosols.^16^ Several studies have shown the link between acidic aerosols and adverse health effects such as respiratory symptoms,^17^ pulmonary dysfunction,^18^ and other epidemiological outcomes.^19^

Reactive oxygen species (ROS), including hydroxyl radicals (^•^OH), superoxide/hydroperoxyl radicals (O_2_^•–^/HO_2_^•^), hydrogen peroxide (H_2_O_2_), and organic radicals, play a central role in multiphase chemistry of atmospheric and physiological processes.^20^ Decomposition of organic hydroperoxides^21^ and peracids^22^ can lead to the formation of ^•^OH, the most reactive form of ROS. The subsequent reactions of ^•^OH with primary or secondary alcohols can lead to the generation of superoxide via decomposition of α-hydroxyperoxy radicals.^23^ Limited studies have investigated the effects of pH on ROS formation. Enami^24^ reported that lower pH promotes the decomposition of α-hydroxyhydroperoxides leading to the formation of H_2_O_2_. Tong et al.^25^ showed significant enhancement in radical formation with highly acidic (pH 0–1) conditions in the mixtures of isoprene SOA and mineral dust, which could be due to enhanced Fenton(-like) reactions in the presence of transition metals in the dust. Our recent study demonstrated substantial formation of organic radicals from iron-facilitated decomposition of organic peroxides contained in SOA in surrogate epithelial lining fluid with a pH of 7.4.^26^ As there have been few systematic investigations of pH effects on ROS formation, it is still highly uncertain how different pH values would affect ROS formation from SOA, and the underlying chemical mechanism is poorly understood.

In this study, we characterized ROS formation from laboratory-generated SOA under three pH range(s): highly acidic (1.0), moderately acidic (2.5–3.5), and neutral (7.4) conditions. We observed that pH impacts yields and the composition of ROS depending on SOA types: isoprene and α-terpineol SOA are found with significantly higher ROS formation at neutral pH; α-pinene, β-pinene, toluene and naphthalene SOA generate more superoxide in acidic conditions. Further, we revealed using model compounds that the radical generation by organic peroxide decomposition can be suppressed under lower pH. In contrast, acidic conditions consistently promote H_2_O_2_ yields from biogenic and aromatic SOA, likely due to the enhanced decomposition of α-hydroxyhydroperoxides and redox cycling by quinone-type compounds, respectively. This work presents the detailed characterization and mechanistic discussion of ROS formation from SOA under different pH values, which have significant implications on atmospheric aerosol processes and oxidative stress.

## Materials and Methods

### Preparation of SOA and Model Compounds

A potential aerosol mass (PAM) reactor^27^ was used to generate SOA particles from ^•^OH photooxidation of isoprene (Sigma-Aldrich, ≥99%), α-terpineol (Arcos Organics, ≥97%), α-pinene (Sigma-Aldrich, 98%), β-pinene (Sigma-Aldrich, ≥99%), toluene (Alfa Aesar, ≥99.7%), and naphthalene (Sigma-Aldrich, ≥99%). Detailed procedures of SOA formation can be found in our recent studies.^23,26^ While the PAM reactor applies high levels of oxidants (i.e., OH^•^ concentration of ∼10^10^ cm^–3^)^28^ with a short reaction time, the PAM-generated SOA have been found to have similar characteristic with ambient and chamber-generated SOA in terms of mass yield, oxidation state, hygroscopicity, and chemical composition with similar mass spectra measured by an Aerodyne ToF AMS.^29−31^ The relative humidity in the PAM reactor was 40–50%. A scanning mobility particle sizer (SMPS, Grimm Aerosol Technik) was used to record the particle size distribution. SOA particles were collected on 47 mm poly(tetrafluoroethylene) (PTFE) filters (Millipore FGLP04700, 0.2 μm pore size) for 60–120 min with average mass loadings of 0.45 ± 0.04, 1.19 ± 0.26, 0.73 ± 0.20, 0.67 ± 0.10, 2.52 ± 0.50, and 0.43 ± 0.12 mg for isoprene, α-terpineol, α-pinene, β-pinene, toluene, and naphthalene SOA, respectively. The filter samples were extracted into 1 mL of 10 mM spin-trap solutions with preadjusted pH (1.0, 2.5–3.5, 7.4) for 7 min. The filters after extraction were dried under nitrogen flow for 10–20 min. The mass difference before and after the extraction was considered as the amount of SOA dissolved in the solution, and an average molar mass of 200 g mol^–1^^21^ was assumed to calculate the SOA molar concentrations in filter extracts. SOA concentrations were in the range of 1.9–2.5, 4.8–7.8, 2.6–5.2, 2.6–4.0, 9.2–15.7, and 1.2–2.5 mM for isoprene, α-terpineol, α-pinene, β-pinene, toluene, and naphthalene SOA, respectively. Two SOA samples were prepared for each pH for the quantification of radicals. The radical formation under different pH values was also quantified using several model compounds including cumene hydroperoxide (Alfa Aesar, 80%), *tert*-butyl hydroperoxide (Sigma-Aldrich, 70%), 5-hydroxy-1,4-naphthoquinone (5-H-1,4-NQ, Sigma-Aldrich, 97%), and ascaridole (MuseChem, >98%).

### pH Control

The SOA-extracted solutions were maintained at highly acidic (pH = 1.0), moderately acidic (pH = 2.5–3.5), or neutral (pH = 7.4) conditions. The highly acidic condition mimics the ambient internally mixed particles of sulfate and organics.^11^ pH was adjusted to 1.0 by adding sulfuric acid (VWR, 95–98%) in the spin-trap solution. The moderately acidic condition is in line with aerosols containing a lower amount of sulfate, biomass burning aerosols,^32^ and the lower end of cloud/fog water droplets.^15^ The original pH of the SOA extracts varied from 2.5 to 3.5, representing a moderately acidic condition. The neutral pH is of interest for cloud droplets as well as the physiological environment upon inhalation and respiratory deposition of SOA.^15^ A phosphate-buffered saline (PBS, Corning, 10×) was used to adjust the pH at 7.4 in the SOA extracts. SOA particles were extracted into a spin-trap solution with preadjusted pH. The pH of the model compounds was maintained the same way for the highly acidic (sulfuric acid) and neutral (PBS) conditions, while a smaller amount of sulfuric acid was used to reach the moderately acidic condition (pH = 3.0). A pH meter (VWR sympHony) was used to measure the pH of the reagents.

### EPR Analysis

A continuous-wave electron paramagnetic resonance (CW–EPR) spectrometer (Bruker, Germany) coupled with a spin-trapping technique was applied to quantify the free-radical formation in the aqueous phase. The free radicals were captured by a spin-trapping agent 5-*tert*-butoxycarbonyl-5-methyl-1-pyrroline-*N*-oxide (BMPO) (Enzo Life Sciences, ≥99%). After particle extraction into 1 mL of 10 mM BMPO solutions, a 50 μL aliquot of the SOA extracts was loaded into a 50 μL capillary tube (VWR) and inserted in the resonator of the EPR spectrometer at 10, 20, 60, 90, and 120 min from the start of aqueous reactions. The parameters for EPR measurements are as follows: a center field of 3515.0 G, a sweep width of 100.0 G, a receiver gain of 30 dB, a modulation amplitude of 1.0 G, a scan number of 10–50, attenuation of 12 dB, a microwave power of 12.6 mW, a modulation frequency of 100 kHz, a microwave frequency of 9.86 GHz, and a conversion time and time constant of 5.12 ms. After obtaining the EPR spectra, SpinFit and SpinCount methods embedded in the Bruker Xenon software were applied to quantify BMPO-radical adducts at each time point.^21^

In addition, an *in situ* UV irradiation system (ER203UV, Bruker, Germany) equipped with a 100 W Hg lamp was used with EPR to characterize the radical formation upon illumination. The lamp was usually warmed up for roughly 10 min before the start of any irradiation experiments. A safety shutter between the lamp and the resonator was used to control the start and stop of irradiation. A liquid light guide focused the light to the EPR resonator where samples were exposed to UV to visible light with a wavelength range of 220–600 nm. To test the pH effect on BMPO trapping efficiencies, 1 mM H_2_O_2_ was mixed with 10 mM BMPO at pH 7.4, 3.0, and 1.0 and then placed into the irradiation system, where H_2_O_2_ can be photolyzed to form ^•^OH. The background spectrum was recorded at the starting point, with the shutter raised after the first EPR measurement was finished (∼1 min). Temporal measurements were then conducted every minute for 10 min to monitor the change in BMPO-OH concentrations over time.

### H_2_O_2_ Measurement

A modified protocol^33^ was applied for the H_2_O_2_ measurement using a fluorometric H_2_O_2_ assay kit (MAK165, Sigma-Aldrich). Detailed procedures of assay preparation can be found in our previous study.^23^ The H_2_O_2_ formation was quantified within 2 h from the preparation of working solutions due to the instability of the probe. A calibration was performed using H_2_O_2_ standard solutions with concentrations ranging from 0.05 to 1.5 μM in PBS to maintain pH at 7.4 (Figure S1). The reaction vials consisted of 2.94 mL sample (Milli-Q water + filter extracts + PBS) and 60 μL working solution. The dilution factors were adjusted for different SOA samples so that the final H_2_O_2_ concentrations would be below 1.5 μM. All H_2_O_2_ measurements were conducted with a filter blank with the same dilution factor as the samples. The addition of working solution to the samples was considered as the start of the reaction, and the measurement was conducted after the reaction vials were incubated at the room temperature of 298 K for 15 min. A spectrofluorophotometer (RF-6000, Shimadzu) was used to measure the fluorescence of the reagents at excitation and emission wavelengths of 540 and 590 nm, respectively.

### Diogenes Chemiluminescence Assay

A Diogenes chemiluminescence assay was applied to quantify the superoxide formation from SOA at neutral pH of 7.4. The reaction products between the Diogenes probe and O_2_^•–^ emit flash chemiluminescence signal proportional to the O_2_^•–^ production rate.^34^ A Microplate Reader (Promega, GloMax) was used to measure the chemiluminescence in a relative light unit (RLU). To convert RLU to O_2_^•–^ production rate, the Diogenes assay was calibrated by the EPR spectrometer using a standardized cell-free O_2_^•–^ generation system—the hypoxanthine (HX) and xanthine oxidase (XO) system. The oxidation process of HX catalyzed by XO can pass electrons to dissolved oxygen to form O_2_^•–^.^35^ A spin probe CMH (1-hydroxy-3-methoxycarbonyl-2,2,5,5-tetramethylpyrrolidine. HCl, Enzo Life Sciences, ≥99%) was used to react with O_2_^•–^ to form nitroxide radical CM^•^ that can be quantified by EPR.^36^ Concentrations of O_2_^•–^ at different time points were obtained by simulating the CM^•^ spectra and then used to calculate the O_2_^•–^ production rate. The detail of the calibration is provided in Supporting Information, and the calibration curve is shown in Figure S2. The O_2_^•–^ production rates from six SOA were measured using the Diogenes method. The SOA samples were directly extracted in 1 mL PBS, after which 135 μL of the SOA extracts were added to Diogenes to initiate the reaction. Two samples were used for each SOA, while a filter blank was used for blank correction. The chemiluminescence measurement was conducted from 1 to 10 min after the extraction was completed. The first data points with the reaction time up to 2 min were used to calculate initial O_2_^•–^ production rates. The chemiluminescence signals were observed to decrease over 10 min, and we integrated O_2_^•–^ production rates to estimate the cumulative O_2_^•–^ production.

## Results and Discussion

### Radical Formation from SOA at Different pH Values

**Figure 1:**
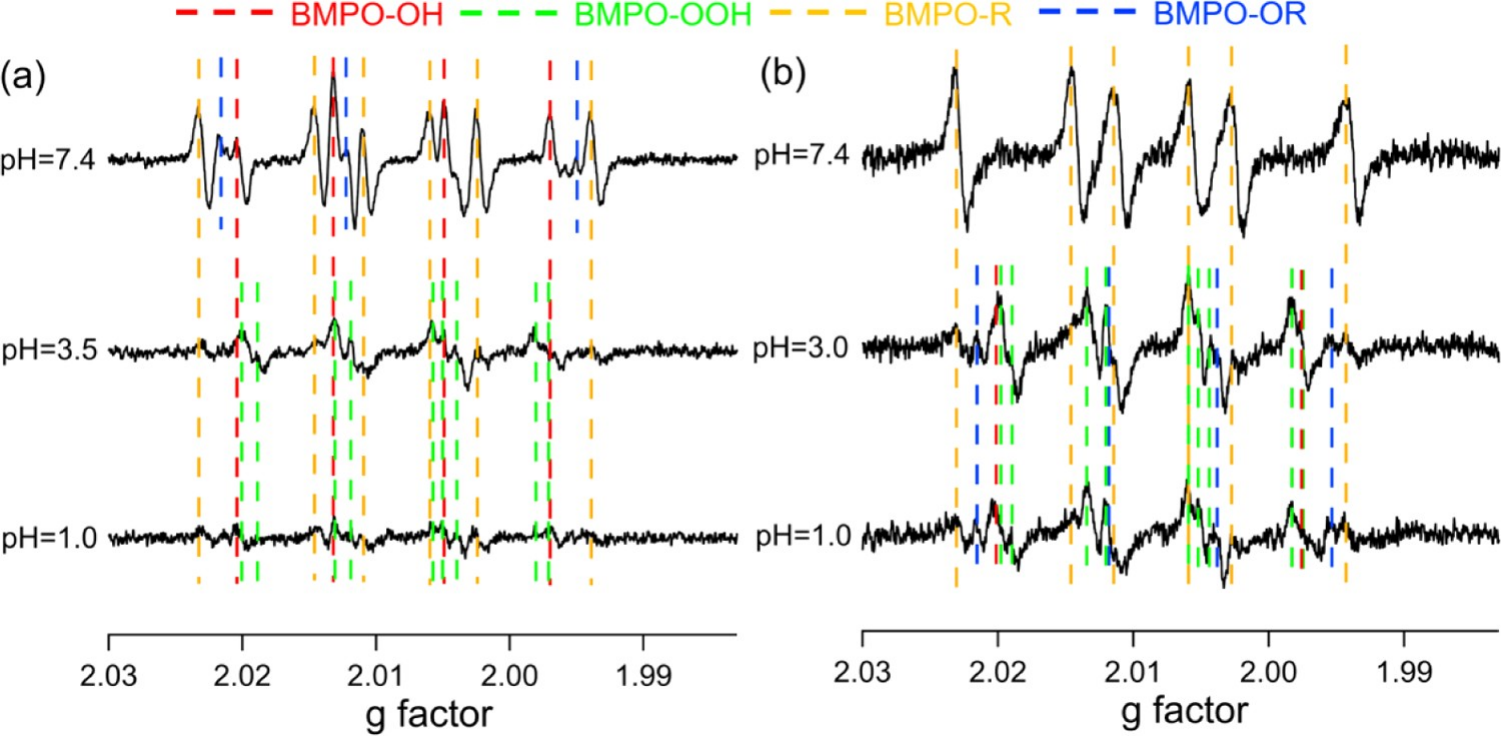
EPR spectra of aqueous extracts of (a) isoprene SOA and (b) α-terpineol SOA at different pH values (7.4, 3.0, and 1.0) in the presence of the spin-trapping agent BMPO. The dashed vertical lines represent different BMPO-radical adducts including OH (red), superoxide (green), and carbon- (orange) and oxygen-centered (blue) organic radicals.

**Figure 2:**
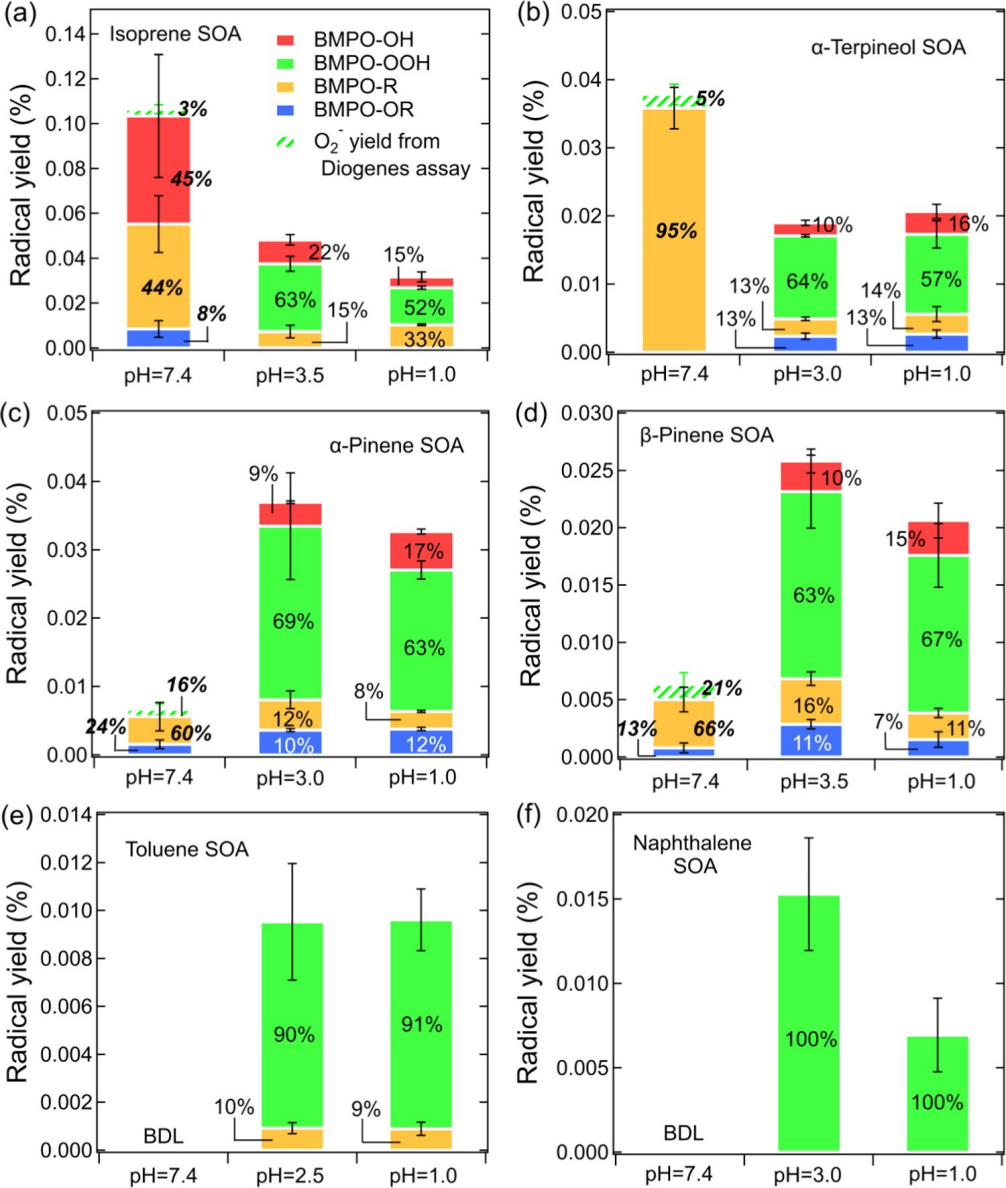
Yields and relative abundance of different radical species from (a) isoprene SOA, (b) α-terpineol SOA, (c) α-pinene SOA, (d) β-pinene SOA, (e) toluene SOA, and (f) naphthalene SOA at different pH values in the presence of BMPO. The solid-colored bars represent BMPO-radical adducts measured by EPR, while the green dashed bars represent superoxide yields estimated from the Diogenes assay. Note the italic bold numbers at pH 7.4 are calculated by combining the results of EPR and the Diogenes assay. The error bars represent the error propagation from the two duplicates in EPR measurements or the Diogenes assay with the uncertainty in SOA mass measurements.

Figure 1a,b shows the observed EPR spectra of the aqueous extracts of isoprene SOA and α-terpineol SOA at different pH values. Each EPR spectrum is composed of several overlapped lines, originating from different radical forms of ROS. The dashed lines indicate the positions of each peak for each type of trapped radicals, including OH (red), superoxide (green), carbon-centered (orange), and oxygen-centered organic radicals (blue). The observed spectra were simulated and deconvoluted to derive the radical yields and relative abundance of different BMPO-radical adducts. As shown in Figure S3, the simulated EPR spectra reproduced the observed spectra very well with small residuals. The solid color bars in Figure 2 show the relative abundance and BMPO-radical yields from SOA generated from six different precursors. As shown in Figure 2a,b, the BMPO-radical adduct yields from isoprene and α-terpineol SOA at neutral pH are significantly enhanced to 0.10 and 0.035% from <0.05 and <0.02% at acidic conditions, respectively. Isoprene SOA at neutral pH generates substantial amounts of ^•^OH (45%) and carbon-centered organic radicals (44%) with a very minor contribution from oxygen-centered organic radicals (8%), while α-terpineol SOA shows dominant carbon-centered radical formation at neutral pH. In comparison, both isoprene and α-terpineol SOA produce O_2_^•–^/HO_2_^•^ (50–60%) predominantly in acidic conditions, while ^•^OH (10–20%) and organic radicals (15–33%) only constitute minor fractions, as consistent with our recent study.^26^ It should be noted that the highly acidic condition (pH = 1.0) does not lead to notable differences in radicals yields and relative abundance compared to the original SOA extracts with moderately acidic conditions (pH = 3.0–3.5).

In addition, we characterized radical formation from α-pinene, β-pinene, toluene, and naphthalene SOA (Figure 2c–f) at different pH values, with the observed EPR spectra shown in Figure S4. α-pinene and β-pinene SOA (Figure 2c,d) show an inverse trend to isoprene and α-terpineol SOA, with substantially lower BMPO-radical yields at neutral pH (<0.01%) compared to acidic conditions (0.02–0.04%). At neutral pH, α-pinene and β-pinene SOA mainly generate low amounts of organic radicals, while the dominant formation of O_2_^•–^/HO_2_^•^ (>60%) is observed at pH 1.0 and 3.0, similar to isoprene and α-terpineol SOA. For aromatic (toluene and naphthalene) SOA (Figure 2e,f), we observed dominant superoxide formation (90–100%) in acidic solutions, whereas no radicals above the detection limit were found at neutral pH.

**Figure 3:**
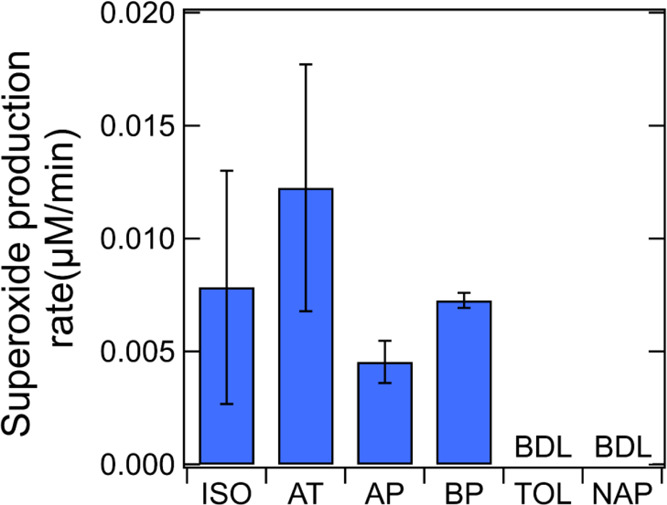
Initial superoxide production rates from SOA generated by isoprene, α-terpineol, α-pinene, β-pinene, toluene, and naphthalene measured by Diogenes chemiluminescence assay at pH 7.4. The error bars represent uncertainties from the two duplicates of SOA in chemiluminescence measurements.

An interesting result as observed from Figure 2 is that no BMPO-OOH (green solid bars) was detected at pH 7.4 for all SOAs, raising a question if the EPR-spin-trap method with BMPO can detect superoxide efficiently at neutral pH. Given the p*K*_a_ of HO_2_^•^ (4.88), the predominant form of superoxide in acidic conditions (pH 3.0 and 1.0) should be HO_2_^•^, whereas it is O_2_^•–^ at neutral pH.^37^ It has been reported that a nitrone spin trap can react with HO_2_^•^ very efficiently (e.g., BMPO + HO_2_^•^ → BMPO-OOH), while the trapping of O_2_^•–^ is a two-step process via the initial addition of O_2_^•–^ to BMPO to form the BMPO-O_2_^–^ adduct followed by protonation by water (or other acidic sources) to form BMPO-OOH.^38^ As a consequence, the overall rate of O_2_^•–^ trapping in neutral conditions can be an order of magnitude slower compared to HO_2_^•^ trapping in acidic conditions.^39^ Hence, we applied the Diogenes chemiluminescence assay which is more sensitive in superoxide measurements at neutral pH (see Supporting Information text and Figure S5). Figure 3 shows the measured initial O_2_^•–^/HO_2_^•^ production rates by SOA at neutral pH. All biogenic (isoprene, α-terpineol, α-pinene, and β-pinene) SOA show positive superoxide production rates, varying from 0.005 to 0.013 μM min^–1^. In contrast, toluene and naphthalene SOA do not generate O_2_^•–^/HO_2_^•^ above the detection limit, as consistent with the EPR-spin-trap method (Figure 2e,f).

Overall, the cross-validation by the Diogenes assay suggests that the superoxide yields at neutral pH may be underestimated by BMPO trapping. Therefore, we estimated the total superoxide production yields by the Diogenes assay at neutral pH, which are added as green dashed bars in Figure 2. For isoprene and α-terpineol SOA, the additional O_2_^•–^ formation at pH 7.4 can further increase the enhancement factors compared to acidic conditions, while the radical yields from α-pinene and β-pinene SOA are still much lower at neutral pH even after considering O_2_^•–^ formation. Both methods confirm that superoxide formation is below the detection limit from toluene and naphthalene SOA at pH 7.4, consolidating that aromatic SOA containing quinone-type compounds mediate redox cycling and O_2_^•–^ formation in a pH-dependent manner favoring stronger acidity.^40^

**Figure 4:**
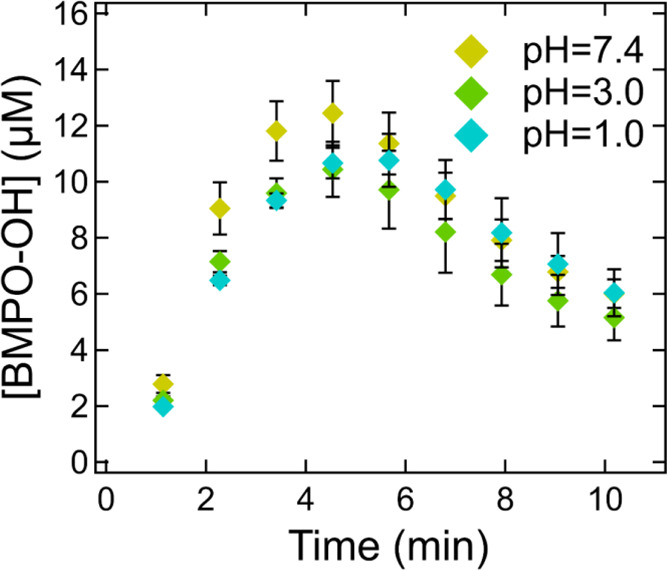
Temporal evolution of concentrations of BMPO-OH adducts from UV–vis irradiation of the mixture of 1 mM H_2_O_2_ and 10 mM BMPO at different pH values. The light was switched on 1 min after the starting point. The error bars represent the uncertainties from the two duplicates in EPR measurements.

We also investigated if pH affects the BMPO trapping efficiency of ^•^OH in the mixtures of 1 mM H_2_O_2_ and 10 mM BMPO under UV–vis irradiation at different pH. Due to the nature of H_2_O_2_ photolysis, the ^•^OH yields should be solely determined by the photon intensity without being affected by pH.^41^ We note that the Fenton reaction (H_2_O_2_ + Fe^2+^), the most common standard system for ^•^OH generation, is unsuitable for the assessment of pH effects on BMPO trapping efficiencies as this reaction is known to be intrinsically affected by pH with higher acidity favoring ^•^OH formation.^42^Figure 4 shows the temporal evolution of BMPO-OH concentrations. For all pH conditions, significant BMPO-OH formation (>10 μM) was observed within 5 min after introducing the irradiation, indicating effective photolysis of H_2_O_2_ and efficient trapping of ^•^OH by BMPO. The sharp decline in [BMPO-OH] after 5 min is likely due to photolytic decay of BMPO-OH. Only marginal differences (<20%) were observed in BMPO-OH concentrations over the course of reactions for different pH conditions, confirming that acidity has minor to negligible effects on the BMPO trapping of ^•^OH. We also speculate that pH effects on BMPO trapping R^•^ and RO^•^ should be trivial as no H^+^ is involved in the reactions. This investigation of the potential pH effects on the trapping efficiencies of BMPO should elicit precaution for future studies using the EPR-spin-trap method especially for detecting superoxide at neutral pH.

### Reaction Mechanisms

**Figure 5:**
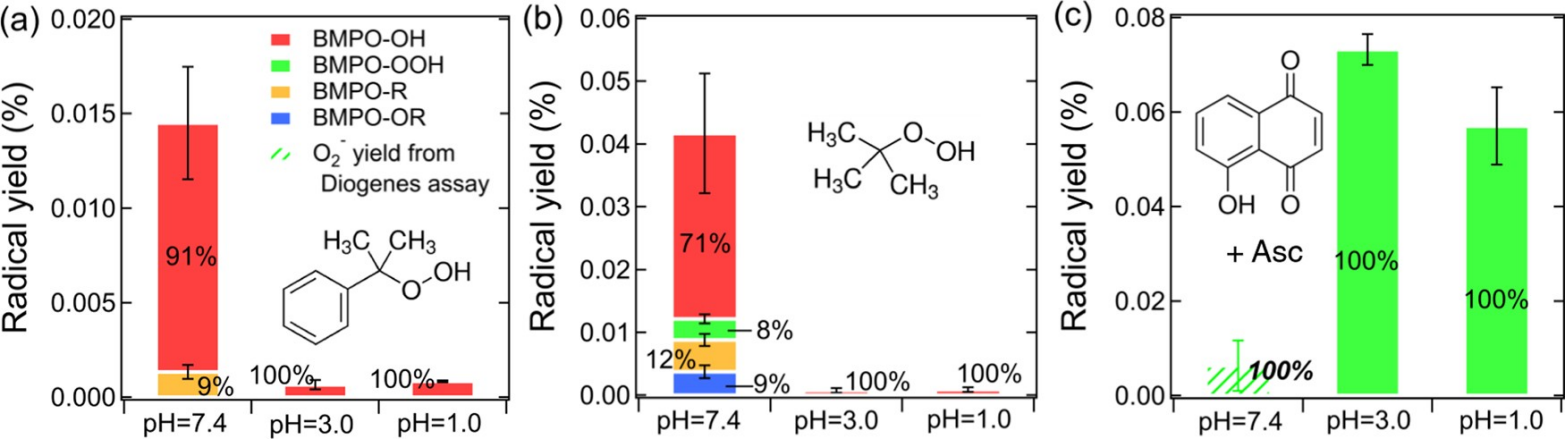
Yields and relative abundance of different radical species from (a) 10 mM cumene hydroperoxide, (b) 10 mM *tert*-butyl hydroperoxide, and (c) mixture of 0.2 mM 5-hydroxy-1,4-naphthoquinone and 0.2 mM ascorbate (Asc) at different pH values in the presence of BMPO. The solid bars represent BMPO-radical adducts measured by EPR, while the green dashed bars represent superoxide yields estimated from the Diogenes assay. The error bars represent the uncertainties from the two duplicates in EPR measurements.

To better understand the pH effects on the ROS formation mechanism from SOA, we measured radical formation from commercially available organic hydroperoxides at different pH values. Figure 5a,b shows predominant ^•^OH formation (70–90%) from 10 mM cumene hydroperoxide (CHP) and *tert*-butyl hydroperoxides at neutral pH, with total radical yields up to 0.014 and 0.04%, respectively. The unimolecular decomposition of labile organic hydroperoxides can lead to ^•^OH formation through the cleavage of the weaker O–O bond.^43,44^ In acidic solutions (pH 3.0 and 1.0), however, both organic hydroperoxides generate much lower ^•^OH (radical yields <0.0009%). While the first-order decomposition of peroxides should be a thermal process depending on temperature instead of pH, it may be suppressed at higher acidity due to the acid-catalyzed rearrangement of alkyl hydroperoxides.^45,46^ Levin et al.^47^ also characterized the kinetics of acid-catalyzed cleavage of CHP leading to phenol and acetone formation, which can take place at or even below room temperature in the presence of sulfuric acid. Further, their study^47^ provides thermodynamic evidence that the thermal decomposition of CHP forming phenol/acetone follows a combined linear-exponential function of sulfuric acid concentration (i.e., pH ≤2.7) at room temperature. This alternative decomposition pathway would lead to alcohol and ketone formation as the end products, involving no radical formation.^48^ A similar mechanism has also been shown for aliphatic alkyl hydroperoxides including *tert*-butyl hydroperoxide.^49^ Therefore, it may partially account for the decreased radical formation by isoprene and α-terpineol SOA at lower pH (Figure 2a,b), although the complex and multifunctionalized nature of organic hydroperoxides in SOA may not be accurately represented by cumene or *tert*-butyl hydroperoxides. The major contribution from ^•^OH by isoprene SOA is likely due to its higher fraction of organic hydroperoxides (3–5%) compared to α-terpineol SOA (1–3%) as predicted by kinetic modeling in our previous study.^23^

Further, the decomposition of organic hydroperoxides is unlikely to account for the exclusive formation of organic radicals by α-terpineol SOA as measured by EPR. Iyer et al.^50^ recently proposed that rapid autoxidation during α-pinene ozonolysis may lead to the formation of endoperoxides through ring opening and hydrogen shift reactions. While common organic peroxides (e.g., *tert*-butyl peroxybenzoate) can be stable under room temperature and do not decompose into organic radicals,^26^ the radical formation from endoperoxides has not been investigated. The decay of ROOR may potentially serve as a plausible channel, as the RO^•^ generated from the O–O cleavage can rapidly undergo isomerization or decomposition to form R^•^.^51,52^ Therefore, we characterized the ROS formation from a commercially available endoperoxide, ascaridole. However, we observed no radicals above the detection limit (Figure S6c), indicating that endoperoxides are not responsible for the organic radical formation from α-terpineol SOA, or the reactivity of ascaridole is lower compared to endoperoxides contained in α-terpineol SOA. Meanwhile, it may be possible that ^•^OH can abstract H from the tertiary alcohol group in α-terpineol SOA to form RO^•^, which undergoes β-scission to form R^•^.^51^ While this mechanism has been demonstrated for the tertiary alcohol group in citric acid,^53^ further studies are warranted as ^•^OH oxidation of monoterpene alcohol has been rarely studied.

Quinones often contained in aromatic SOA are well known to induce superoxide formation: in the presence of an electron donor, quinones can be reduced to semiquinone radicals which can further react with dissolved oxygen to form superoxide.^54,55^ The pH dependence of the quinone redox cycling has been rarely discussed in the context of ambient PM, so we measured radical formation in the mixture of 0.2 mM 5-hydroxy-1,4-naphthoquinone (5-H-1,4-NQ) and 0.2 mM ascorbate. Note that 5-H-1,4-NQ alone did not generate radicals above the detection limit. Figure 5c shows significantly higher superoxide production at lower pH. It has been demonstrated that the quinone–hydroquinone couple has a redox potential dependent on pH in a straightforward Nernstian manner,^40^ which follows that increasing pH causes a decrease in the redox potential.^56^ This provides a thermodynamic explanation on favorable O_2_^•–^/HO_2_^•^ formation through stronger quinone redox cycling in acidic conditions compared to neutral pH (Figure 2e,f). It has been shown that hydroquinones can be unstable at physiological pH, undergoing autoxidation to form semiquinone radicals and quinones with concomitant generation of O_2_^•–^ and H_2_O_2_.^57^ Further studies are necessary to evaluate the relevance of such pathways especially for SOA generated from phenolics such as catechol and cresol.^58,59^

### H_2_O_2_ Formation from SOA at Different pH Values

**Figure 6:**
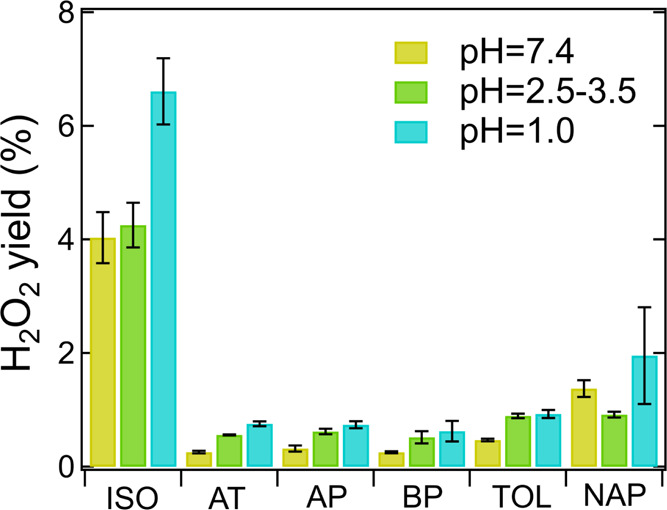
H_2_O_2_ yields from aqueous reactions of isoprene SOA (ISO), α-terpineol SOA (AT), α-pinene SOA (AP), β-pinene SOA (BP), toluene SOA (TOL), and naphthalene SOA (NAP) at different pH values. The actual pH of each SOA in the 2.5–3.5 range corresponds to those in Figure 2. The error bars represent the error propagation from the two duplicates in fluorescence measurements and the uncertainty in SOA mass measurements.

In addition to radicals, we characterized H_2_O_2_ yields from all SOA at different pH values, as shown in Figure 6. Overall, higher H_2_O_2_ yields are consistently observed for all SOA as pH decreases from 7.4 to 1.0, with the enhancement factors varying from 1.5 to 3. This is in good agreement with Wang et al.,^60^ who observed that H_2_O_2_ generation by α-pinene, β-pinene, and toluene SOA increased by a factor of 1.5, 2.4, and 1.75, respectively, when pH decreased from 7.5 to 3.5. Isoprene SOA shows significantly higher yields of H_2_O_2_ (4.0–6.6%) compared to other SOA (<2.0%) with the H_2_O_2_ level (4.2%) in the original extract (pH 3.5) in excellent consistency with our previous study (4.3 ± 0.4%).^23^ Naphthalene SOA shows the second-highest H_2_O_2_ yields (1.4–2.0%), which is comparable with Liu et al.^61^ (1.9–2.5%). Qiu et al.^62^ recently proposed that the decomposition of α-hydroxyalkyl-hydroperoxides (α-HHs) is a proton-catalyzed process associated with H_2_O_2_ formation, which is a highly plausible mechanism accounting for the elevated H_2_O_2_ yields from biogenic SOA. They showed that the decay rates of α-HHs derived from α-terpineol increase drastically from 0.29 × 10^–3^ to 12 × 10^–3^ s^–1^ when pH decreases from 5.7 to 3.3.^62^ For toluene and naphthalene SOA, the enhanced superoxide formation with higher acidity may subsequently lead to H_2_O_2_ yields because O_2_^•–^ is known as an important precursor of H_2_O_2_.^64^ Given the low O_2_^•–^ formation (Figure 2) but high H_2_O_2_ yields from naphthalene SOA, additional H_2_O_2_ sources could be important including decomposition of hydroxyhydroperoxides,^61^ which may account for significant fractions in naphthalene SOA.^65^

## Implications

This work provides a detailed characterization of pH effects on ROS formation from various SOA and probes into the underlying mechanisms in acidic versus physiological pH. In atmospheric aerosols, acidity plays a critical role in chemical transformation and composition by regulating acid-catalyzed particle-phase reactions. A primary mechanism is through acid-catalyzed aldehyde and carbonyl reactions including protonation, hydration, and addition of alcohol,^66^ which can contribute to high aerosol yields due to oligomerization and aldol condensation.^67,68^ It has also been well studied that higher acidity can enhance isoprene SOA concentrations by triggering ring opening of epoxydiols followed by the nucleophilic addition of inorganic sulfate.^69,70^ In comparison, acid-catalyzed reactions of organic peroxides have been less discussed despite their significance in aqueous-phase radical formation. A recent study demonstrated that carboxylic acid can catalyze the reaction between hydroperoxides and aldehydes to form peroxyhemiacetals.^71^ In addition, Hu et al.^72^ reported that the decomposition of α-alkoxyalkyl-hydroperoxides can be enhanced at lower pH involving no radical formation. Thus, the complex nature of SOA can alter the ROS formation capacity of organic peroxides under acidic conditions. These aspects should be considered along with the acid-catalyzed rearrangement of hydroperoxides to better understand ROS formation from SOA.

In analogy to ROOH (e.g., CHP), α-HHs undergo acid-catalyzed decomposition forming carbonyls and H_2_O_2_, as shown by substantially higher H_2_O_2_ yields in acidic conditions observed in this work. α-HHs can originate from hydrolysis of Criegee intermediates^62^ or ^•^OH oxidation of alcohols to form α-hydroxyalkyl radicals followed by O_2_ addition and HO_2_^•^ termination.^63^ A very recent study indicated the dominant contribution of decomposition/hydrolysis of organic peroxides to the condensed-phase H_2_O_2_, whereas the partitioning of the gas-phase H_2_O_2_ was negligible.^73^ In the presence of transition metals, H_2_O_2_ can be further converted to much more reactive ^•^OH and induce the formation of highly oxygenated species and chemical aging.^74^ Recent field measurements revealed that elevated H_2_O_2_ concentrations are associated with haze events, and H_2_O_2_ oxidation may act as the primary pathway for sulfate formation.^75,76^ Therefore, our work highlights the importance of acidity in altering the ROS formation yield and composition and the acidity should be considered for further investigations of ROS formation from SOA.

Inhalation and deposition of organic aerosols can lead to oxidative stress by the formed ROS at physiological pH. H_2_O_2_ yields from SOA are shown to be 25–100 times and 5–8 times higher than the total radical yields in acidic and neutral conditions, respectively, which indicates H_2_O_2_ as the most abundant exogenous ROS in ambient PM especially considering its much longer lifetime. Under neutral conditions, organic hydroperoxides can preferably undergo unimolecular decomposition to generate highly reactive ^•^OH radicals, which can initiate a cascade of reactions to propagate further radical formation^74^ as well as directly attack biological components to induce pathological processes such as lipid peroxidation.^77^ The formed organic radicals can be persistent even in the presence of antioxidants,^26^ although their capacity to cause oxidative potential still warrants further studies. While this study used the PAM reactor to generate SOA, further experiments are necessary with SOA generated in an environmental chamber that applies lower VOC and oxidant concentrations as well as with organic particles collected from the ambient atmosphere to consolidate the atmospheric and health relevance of acidity effects on ROS formation by SOA.

## Supporting Information Available

Calibration of the fluorometric H_2_O_2_ assay; calibration of Diogenes chemiluminescence assay; observed and deconvluted EPR spectra from isoprene and α-terpineol SOA at different pH values; EPR spectra of α-pinene SOA, β-pinene SOA, toluene SOA, and naphthalene SOA at different pH values; superoxide measurement at neutral pH by EPR and chemiluminescence; and EPR spectra of model compounds (PDF)
