# Supplementary material for: Effects of Acidity on Reactive Oxygen Species Formation from Secondary Organic Aerosols
Source: ACS Environ Au. 2022 Apr 29;2(4):336–45. doi: 10.1021/acsenvironau.2c00018 (PMC9342606; doi:10.1021/acsenvironau.2c00018)
Supplement: Supplementary file 1 — vg2c00018_si_001.pdf [file vg2c00018_si_001.pdf]

Supporting Information

Effects of Acidity on Reactive Oxygen Species Formation from Secondary Organic Aerosols

Jinlai Wei[1], Ting Fang[1], Manabu Shiraiwa[1,*]

[1] Department of Chemistry, University of California, Irvine, CA, 92697-2025, USA

* m.shiraiwa@uci.edu

Calibration of Diogenes Chemiluminescence Assay

We used the HX/XO system to perform the calibration of the Diogenes chemiluminescence assay. The concentration of HX was fixed at 250 μM and XO was varied from 0 – 1.0 mU mL^{-1} (U as the enzyme unit in μmol min^{-1}). First, XO and probe (either 50 μL Diogenes or 15 μL 3 mM CMH in 200 μL reaction vials) were mixed in PBS, which generated negligible chemiluminescence or $CM^{\bullet}$ signal. Next, HX was added to initiate the reaction to produce $O_2^{\bullet-}$. Chemiluminescence and EPR measurements were then conducted within 1 to 6 minutes of reaction. In control groups, 15 μL superoxide dismutase (SOD) was used to suppress the signals to below detection limit, suggesting that the signals without SOD were due to $O_2^{\bullet-}$ formation. The sample data were corrected with the SOD groups, which involves the propagation of standard deviation. Figure S2 shows a linear relationship (R^2 = 0.97) between $O_2^{\bullet-}$ production rates calculated from the EPR-CMH method and RLU from chemiluminescence. The slope from the linear regressions was used to convert the Diogenes chemiluminescence signals to $O_2^{\bullet-}$ production rates in the unit of μM min^{-1}.

Superoxide Measurement at Neutral pH by EPR and Chemiluminescence

Using the same standard system of superoxide generation – HX/XO, we compared the sensitivities of the EPR-spin trapping technique and the Diogenes assay at neutral pH. Figure S5 shows the accumulation of BMPO-OOH adducts from 250 μM HX and 1.0 munit/mL XO, reaching 1.37 μM in 60 minutes. The XO concentration of 1.0 munit/mL corresponds to the highest point in Fig. S1, which translates into a superoxide production rate of ~ 0.3 μM/min. Thus, it can be roughly estimated that the total superoxide produced over 60 minutes should be ~ 18 μM, which is more than one order of magnitude greater compared to the results of the EPR-BMPO method (1.37 μM). In addition, the Diogenes assay is capable of superoxide measurements at neutral pH for biogenic SOA (Fig. 3), while we observed no superoxide above detection limit from the EPR-BMPO method (Fig.2a-d). Therefore, it can be concluded that the Diogenes assay is substantially more sensitive by about one order of magnitude in superoxide measurements compared to the EPR-BMPO method at neutral pH.

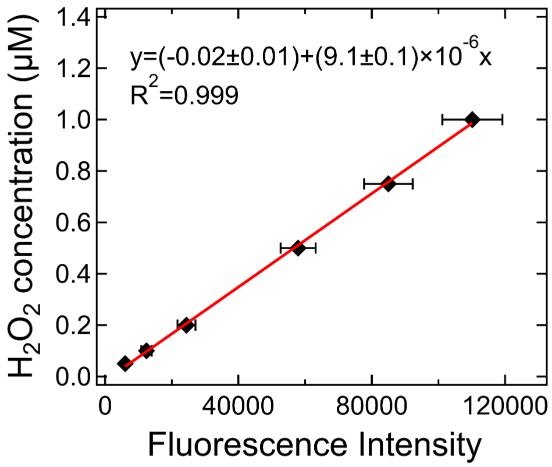

Figure S1. Calibration of the flurometric H_2O_2 assay with 0.05 – 1.5 μM H_2O_2 in phosphate buffer saline (PBS). The fluorescence data were shown as the mean values of two independent experiments.

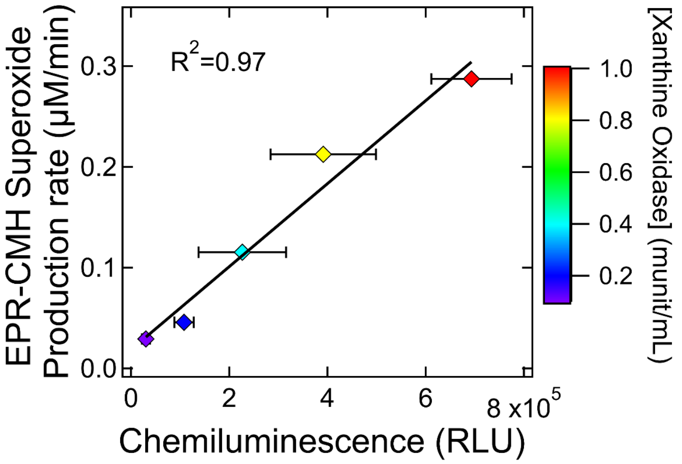

Figure S2. Calibration of Diogenes chemiluminescence by the superoxide production rate determined from the EPR-spin probe method with 250 μM HX and 0 – 1.0 munit/mL XO. The chemiluminescence data were shown as the mean values of two independent experiments.

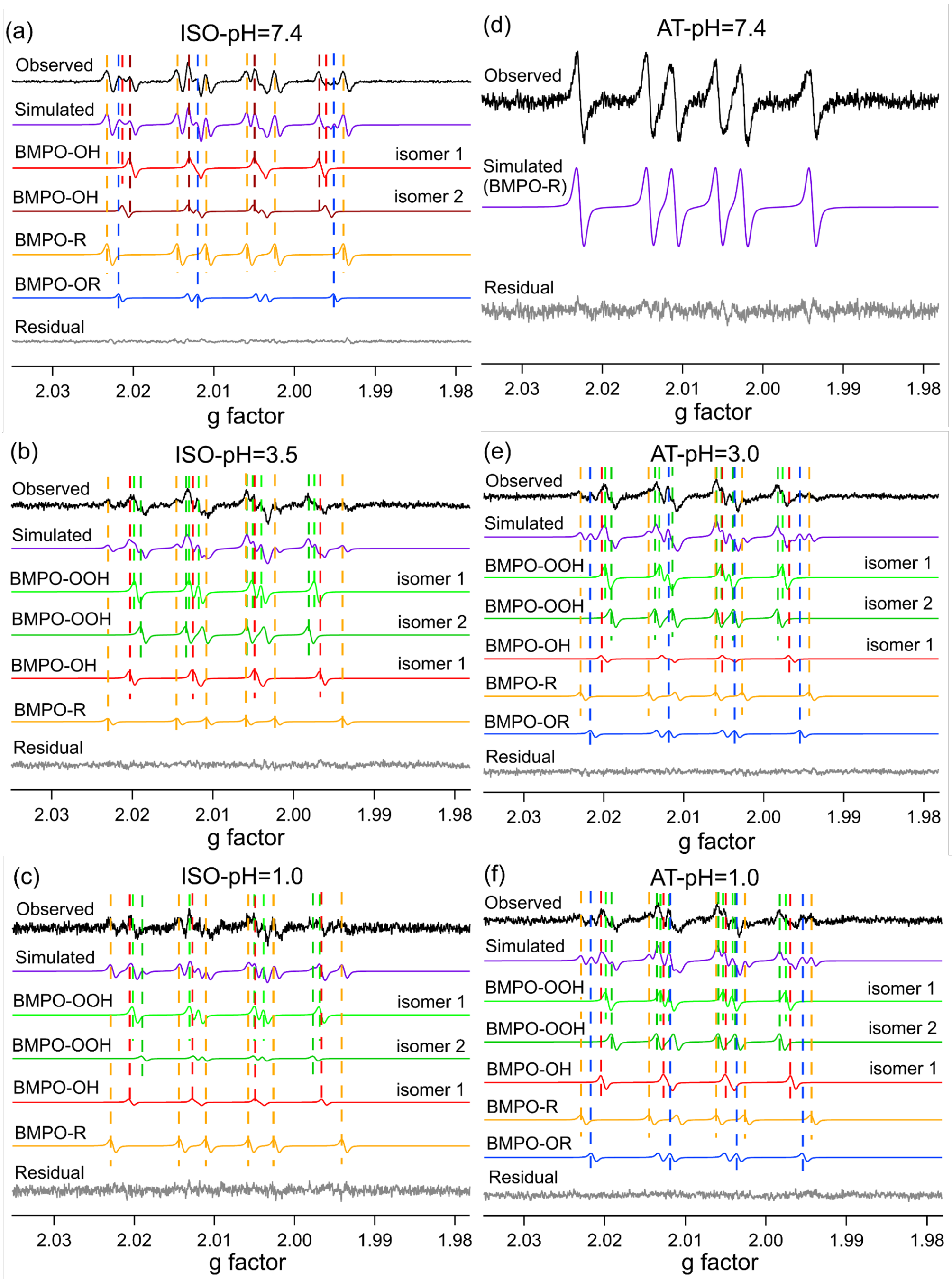

Figure S3. EPR-spectra of BMPO-radical adducts from aqueous reactions of isoprene SOA with (a) pH = 7.4, (b) pH = 3.5, (c) pH = 1.0 and α-terpineol SOA with (d) pH = 7.4, (e) pH = 3.0 and (f) pH = 1.0. The observed spectra (black) are simulated (purple) and deconvoluted into BMPO-OH isomer 1 (red), BMPO-OH isomer 2 (brown), BMPO-OOH isomer 1 (light green), BMPO-OOH isomer 2 (dark green), BMPO-R (yellow), and BMPO-OR (blue). Residual (grey) denotes the difference of observed and simulated spectra. The two isomers of BMPO-OH and BMPO-OOH represent the trans of cis structures of the -OH and -OOH groups, respectively.

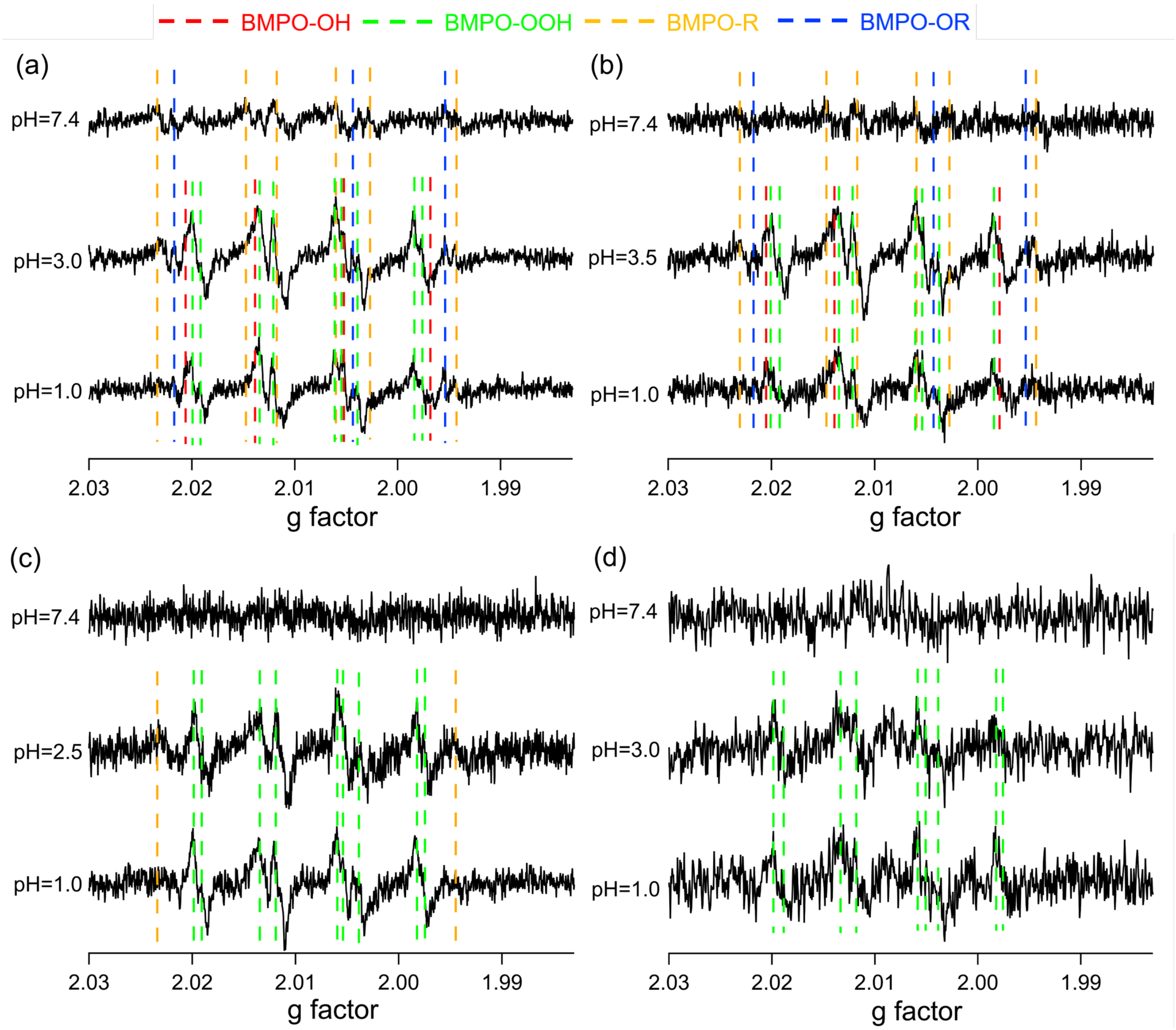

Figure S4. EPR spectra of (a) α-pinene SOA, (b) β-pinene SOA, (c) toluene SOA and (d) naphthalene SOA at different pH in the presence of spin-trapping agent BMPO. The dashed vertical lines represent different BMPO-radical adducts.

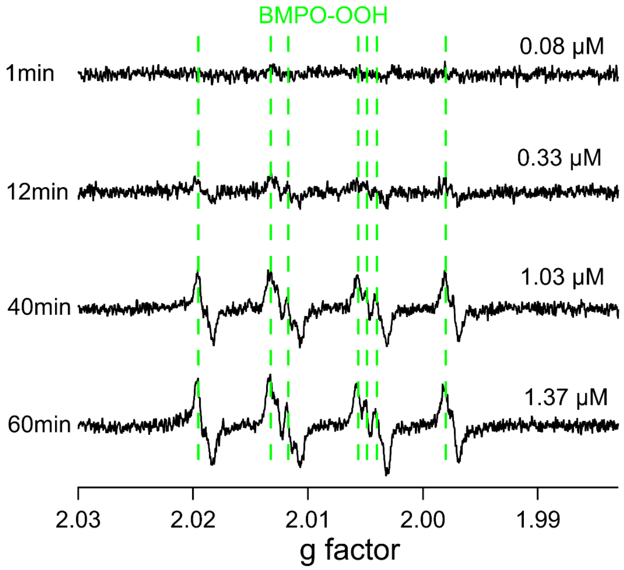

Figure S5. EPR spectra from the mixture of 250 μM HX, 1.0 munit/mL XO and 10 mM BMPO in phosphate buffer saline (PBS) over different time points. The numbers to the right of each spectrum denote the concentrations of BMPO-OOH at each time point. Note that the BMPO-OOH concentrations after the addition of SOD are below detection limit; thus their EPR spectra are not shown here.

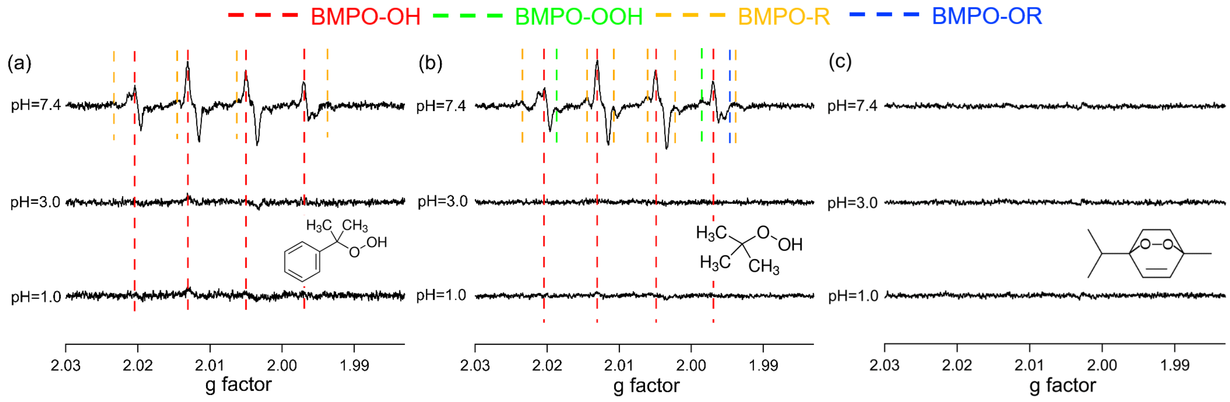

Figure S6. EPR spectra of 10 mM (a) cumene hydroperoxide, (b) *tert*-butyl hydroperoxide and (c) ascaridole at different pH(s) in the presence of BMPO. The dashed vertical lines represent different BMPO-radical adducts.
